# Single-Step Extraction Coupled with Targeted HILIC-MS/MS Approach for Comprehensive Analysis of Human Plasma Lipidome and Polar Metabolome

**DOI:** 10.3390/metabo10120495

**Published:** 2020-12-02

**Authors:** Jessica Medina, Vera van der Velpen, Tony Teav, Yann Guitton, Hector Gallart-Ayala, Julijana Ivanisevic

**Affiliations:** 1 Metabolomics Platform, Faculty of Biology and Medicine, University of Lausanne, CH-1005 Lausanne, Switzerland; Jessica.medina@unil.ch (J.M.); vera.vandervelpen@unil.ch (V.v.d.V.); tony.teav@unil.ch (T.T.); 2 Laboratoire d’Etude des Résidus et Contaminants dans les Aliments (LABERCA), Oniris, INRAE, F-44307 Nantes, France; yann.guitton@oniris-nantes.fr

**Keywords:** sample preparation, extraction, lipidomics, metabolomics, LC-MS/MS, human plasma

## Abstract

Expanding metabolome coverage to include complex lipids and polar metabolites is essential in the generation of well-founded hypotheses in biological assays. Traditionally, lipid extraction is performed by liquid-liquid extraction using either methyl-tert-butyl ether (MTBE) or chloroform, and polar metabolite extraction using methanol. Here, we evaluated the performance of single-step sample preparation methods for simultaneous extraction of the complex lipidome and polar metabolome from human plasma. The method performance was evaluated using high-coverage Hydrophilic Interaction Liquid Chromatography-ESI coupled to tandem mass spectrometry (HILIC-ESI-MS/MS) methodology targeting a panel of 1159 lipids and 374 polar metabolites. The criteria used for method evaluation comprised protein precipitation efficiency, and relative MS signal abundance and repeatability of detectable lipid and polar metabolites in human plasma. Among the tested methods, the isopropanol (IPA) and 1-butanol:methanol (BUME) mixtures were selected as the best compromises for the simultaneous extraction of complex lipids and polar metabolites, allowing for the detection of 584 lipid species and 116 polar metabolites. The extraction with IPA showed the greatest reproducibility with the highest number of lipid species detected with the coefficient of variation (CV) < 30%. Besides this difference, both IPA and BUME allowed for the high-throughput extraction and reproducible measurement of a large panel of complex lipids and polar metabolites, thus warranting their application in large-scale human population studies.

## 1. Introduction

Blood plasma is one of the most commonly used biofluids for metabolic phenotyping, specifically in human population studies. This is mainly due to its easy access with minimally invasive sampling and the ability of its metabolic profile to inform about the systemic physiological status. Blood has a vital physiological role in the transport of circulating metabolites; it supplies tissues with nutrients and oxygen, and it carries away the metabolic by-products and carbon dioxide. Human plasma contains a wide diversity of low molecular weight metabolites, including amino acids, other organic acids, fatty acids, sugars, and complex lipids [1]. Lipids represent more than 70% of plasma metabolome diversity and so far, more than 600 distinct lipid molecular species have been detected in human plasma [2]. These lipids can be classified, depending on their chemical structure, according to Lipid Maps consortium (http://www.lipidmaps.org) into sphingolipids, glycerophospholipids, fatty acids, glycerolipids, sterols, and prenols [3,4]. Their main functions comprise energy storage, signal transduction, and plasma membrane and organelles constituents, thus enabling the cell growth, proliferation, activation, and apoptosis [5]. When compared to total lipid content, the total polar metabolite content (comprising mainly carbohydrates, amino acids, and other organic acids), represents a minor part of plasma metabolome [2]. These polar metabolites are known to regulate the intracellular energy metabolism, including glucose, lipid, and amino acid oxidation [6,7]. The concentrations of endogenous, polar, and lipid metabolites in plasma are determined by cellular activity, and additional internal (e.g., microbiome, inflammation) and external factors (e.g., diet, physical activity, medication, environmental exposures, etc.). Therefore, the recorded changes in respective metabolite concentrations can serve as a readout of cellular biochemical activity and its response to changes in the environment. Hence, an approach to extract and measure the broadest possible range of lipids and polar metabolites in human plasma is fundamental for better understanding of metabolic changes in different physiological conditions [8,9].

Despite having the same analytical principle, lipidomics and metabolomicshave been considered as distinct technological approaches, which is mainly due to the application of different sample preparation and data acquisition protocols. Traditionally, polar metabolite extraction is performed in a single-step using methanol (MeOH) [10,11,12], and lipid extraction using biphasic Folch or Bligh and Dyer protocols with a mixture of methanol, chloroform, or dichloromethane, and water [13,14]. The main advantage of these protocols is the recovery of a wide range of lipid classes in the organic phase and polar metabolites in the aqueous phase and, therefore, their applicability for multicomponent analyses [4,15,16]. However, the main drawback constitutes the reproducible recovery of the bottom organic phase when analyzing large sample sets. Consequently, in order to overcome this issue, chloroform and dichloromethane were replaced by methyl-tert-butyl ether (MTBE) in the Matyash method, where lipids are recovered in the upper organic phase [17]. Still, the reproducible separation of organic and aqueous phase remains laborious and can introduce a bias to the measurement accuracy of some amphiphilic metabolites partitioned between these two phases [15].

Recently, the Alshehry method or 1-BUtanol:MEthanol mixture (1:1, BUME) and isopropanol (IPA, 100%) were introduced as an efficient single-step alternative for complex lipid extraction, also suitable for automation [18,19,20]. Single-step methods are advantageous because they require minimal sample handling to ensure reproducible sample preparation in large-scale studies. In addition, they allow for the simultaneous multicomponent extraction and sample cleaning (deproteinization). Systematic evaluations of single-step *versus* traditionally used biphasic extractions are sparse and the potential of these straightforward protocols to simultaneously extract complex lipids and polar metabolites has not yet been assessed. So far, sample preparation using the Matyash biphasic protocol was evaluated as the best compromise for the comprehensive extraction of complex lipids and an adequate yield of polar metabolites [21,22].

In this work, we demonstrate the performance of two single-step sample preparation methods, i.e., using BUME and IPA, for the simultaneous extraction of complex lipids and polar metabolites from human plasma. The extraction method evaluation was based on protein removal efficiency, relative signal intensity and signal reproducibility of the complex lipid and polar metabolite profiles acquired by high-coverage targeted HILIC-MS/MS. The complex lipidome and polar metabolome coverage was assessed for both BUME and IPA by considering the signal reproducibility (the coefficient of variation (CV) < 30%) as a prerequisite for the measurement accuracy and precision, rather than only focusing on the diversity of detectable metabolome and lipidome.

## 2. Results

### 2.1. Sample Preparation Methods and Evaluation Workflow

**Figure 1:**
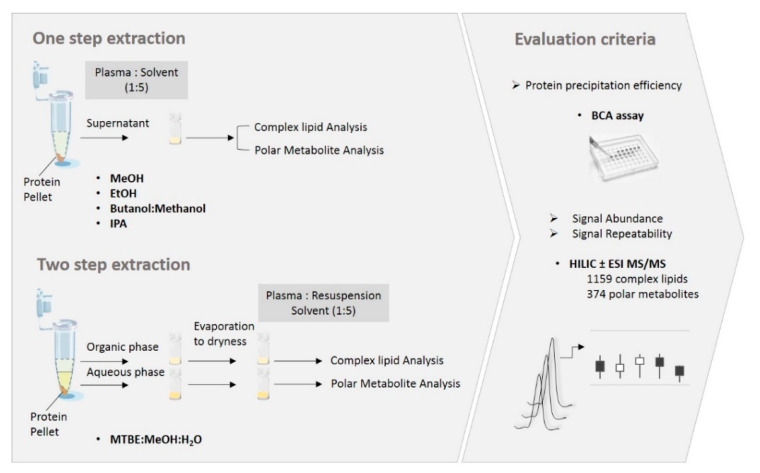
Sample preparation and evaluation workflow. The performance of single step extraction methods, including methanol (MeOH), ethanol (EtOH), BUtanol:MEthanol (BUME), and isopropanol (IPA), was compared to the solvent mixture considered as the best compromise, i.e., MTBE:MeOH:H_2_O for complex lipid and MeOH for polar metabolite extraction. The criteria used for evaluation of solvent performance are shown on the right side of the figure. For the repeatability experiments, the extraction was done by four independent operators each performing five technical replicates per extraction protocol (*n* = 4 operators x 5 replicates).

The ability of single-step methods to simultaneously extract complex lipids and polar metabolites was evaluated using relative abundance and repeatability of metabolite signal, against the commonly applied protocols, a biphasic extraction with MTBE for lipids, and a single-step MeOH for polar metabolites (Figure 1). The extracts were analyzed with high-coverage targeted profiling using HILIC-MS/MS in positive and in negative ionization mode (see Materials and Methods). These methods targeted a total of 1159 lipids from five different lipid classes (sphingolipids, cholesterol esters, glycerolipids, glycerophospholipids, and free fatty acids, Tables S1 and S2), and 374 polar metabolites from 12 different classes (amino acids and their derivatives, carboxylic acids, acylcarnitines, nucleotides, etc., Tables S3 and S4). The classification of polar metabolites was based on the Human Metabolome Database (HMDB) while the complex lipids were classified according to LipidMaps [4,23]. The protein precipitation efficeincy, the relative abundance and the coefficient of variation of each detected metabolite were used to evaluate the extraction performance for each lipid and polar metabolite class (Figure 1).

Firstly, we examined the performance of single-step methods, using MeOH, ethanol (EtOH), BUME, and IPA, to extract lipids. Following the lipid extraction, the protein precipitation efficiency of these four solvents was evaluated by measuring the total protein content in the supernatant and in the pellet (see Materials and Methods section for more details). The equivalent to 95% of protein removal was achieved with all four methods (See Table S5).

Secondly, the relative signal abundance of the entire panel of detected lipid species was evaluated in all four plasma extracts (Figure S1). Methanol demonstrated the poorest performance for complex lipid extraction, with the exception of lysophosphatidylcholines (LPCs) and lysophosphatidylinositols (LPIs). A significantly lower signal for several lipid classes (i.e., sphingomyelins, fatty acids, and lysophospholipids) was also obtained from ethanol extracts, when compared to IPA and BUME. Therefore, MeOH and EtOH were excluded from further evaluation as non-suitable or less performant for complex lipid extraction, respectively. The best candidates, BUME and IPA, were further evaluated against biphasic Matyash method, for their capacity to reproducibly extract lipids and polar metabolites.

### 2.2. Relative Signal Abundance

BUME and IPA extractions were selected as the best single-step methods when considering the relative abundance of extracted lipids. Therefore, they were further evaluated for lipid and polar metabolite extraction against commonly used biphasic extraction MTBE:MeOH:H_2_O in lipidomics studies, and MeOH in polar metabolome studies.

**Figure 2:**
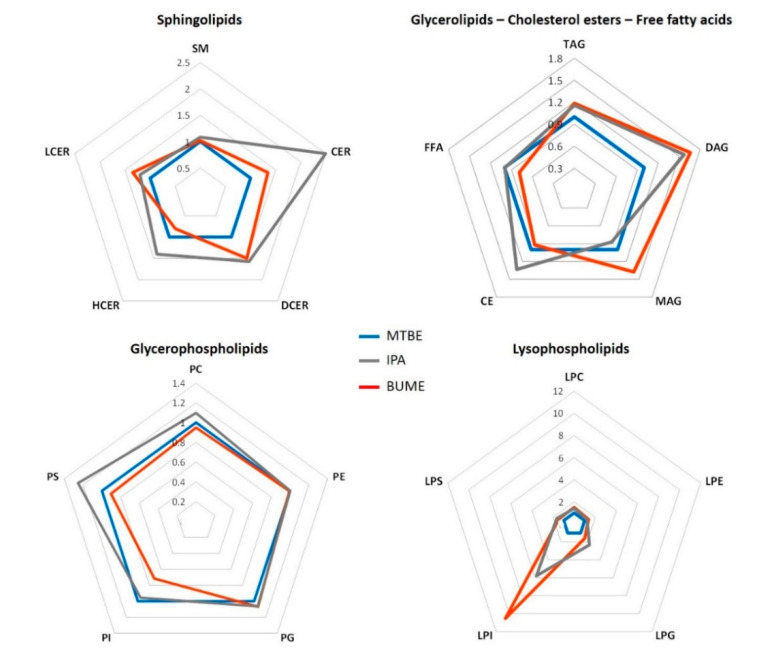
Relative signal abundance (per lipid class) to signal recovered with biphasic extraction with methyl-tert-butyl ether (MTBE). Values indicated on spider plots represent the relative signal abundance (or fold change) for each single-step method, using IPA or BUME, to the reference MTBE extract. For statistical significance see Figure S2 in the Supplementary Information (data are given in Tables S7 and S8). Lipid classification abbreviations: SM—sphingomyelin, CER—ceramides, DCER—dihydroceramides, LCER—lactosylceramiedes, HCER—hexosylceramides, MAG—monoacylglycerol, DAG—diacylglycerol, TAG—triacylglycerol, CE—cholesterol esters, FFA—free fatty acids, PI—phosphatidylinositol, PC—phosphatidylcholine, PG—phosphatidylglycerol, PE—phosphatidylethanolamine, PS—phosphatidylserine, LPC—lysophosphatidylcholine, LPG—lysophosphatidylglycerol, LPE—lysophosphatidylethanolamine, LPI—lysophosphatidylinositol, LPS—lysophosphatidylserine.

**Figure 3:**
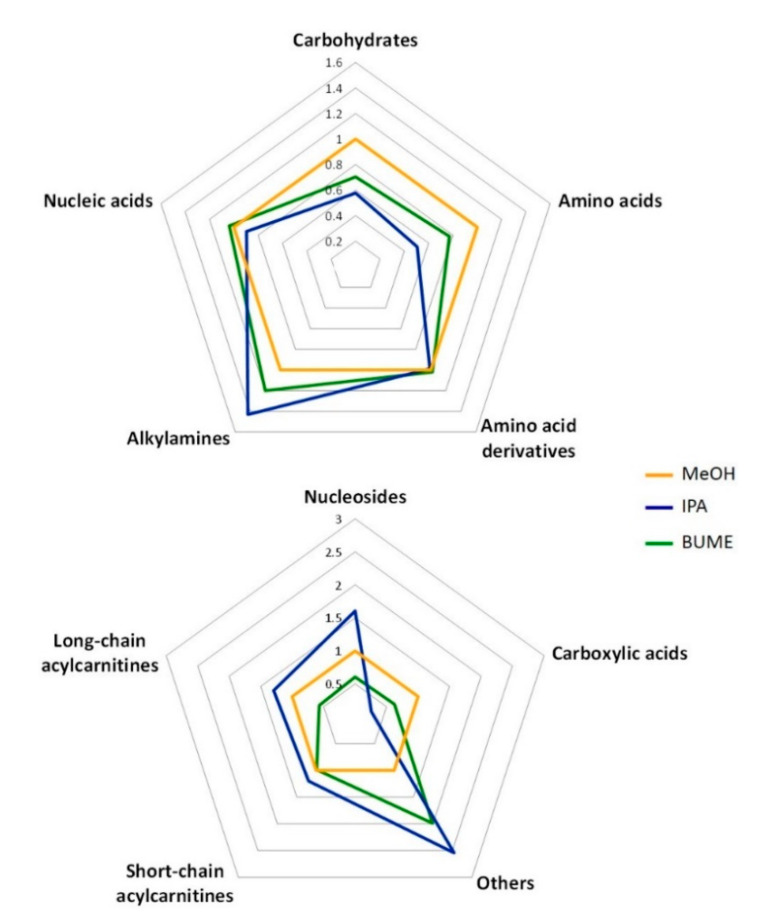
The relative abundance of polar metabolite classes in IPA and BUME extracts as compared to MeOH plasma extract. Values indicated on spider plots represent the relative signal abundance (or fold change) for each single-step method, using IPA or BUME, to the reference MeOH extract. For statistical significance, see Figure S7 in the Supplementary Information (data are provided in Table S9). For the comparison with aqueous phase from Matyash method see Figure S8. The class “Others”, i.e., other polar metabolites comprise glycocholate, hydroquinone, hydroxyphenyllactate, pyridoxine, salsolinol, trigonelline, and tryptamine (Table S6). Carboxylic acids comprised mono-, di-, and tri-carboxylic acids.

Pooled plasma samples were extracted, and the supernatant of each solvent was evaluated per metabolite and lipid class covered by targeted HILIC-MS/MS methods. To acquire the lipid and polar metabolite profiles, the metabolite separation was based on the interaction with the HILIC stationary phase: amide-based for complex lipids in positive and in negative ionization mode, and amide-based and with zwitterionic exchange for polar metabolites in positive and in negative ionization mode, respectively [11]. The extraction capacity of each solvent was evaluated by the relative abundance of each lipid and metabolite class to the average signal abundance obtained by the reference solvents (MTBE mixture for lipids and MeOH for polar metabolites, Figure 2, Figure 3 and Figures S2–S4).

#### 2.2.1. Complex Lipid Profile

Initially, twenty lipid sub-classes were targeted by monitoring 1159 characteristic MRM transitions combining positive and negative ionization mode. Following the filtering of noisy signals, the detectable lipidome in human plasma was resumed to 584 lipid species. In the positive mode, 324 lipids were detected, including sphingomyelins (SM), ceramides (CER), dihydroceramides (DCer), lactosylceramides (LCer), hexosylceramides (HCer), mono, di, triacylglycerols (MAG, DAG, TAG, respectively), and cholesterol esters (CE). In negative mode, 260 lipids were detected, comprising phosphatidylinositols (PI), phosphatidylcholines (PC), phosphatidylglycerols (PG), phosphatidylethanolamines (PE), phosphatidylserines (PS), and their lysophospholipid analogues (LPC, LPG, LPE, LPI, LPS), and free fatty acids (FFA).

The signal abundance recovered (with each extraction method) was evaluated by relative comparison to the average signal of the reference extract, in this case, the MTBE for lipid extraction. Lipid species that were representative of all different lipid classes were successfully extracted by all three solvents tested, with the exception of LPIs, for which the signal in the MTBE extract was close to the limit of detection, as shown in Figure 2. Among sphingolipids, the highest signal was obtained with IPA and BUME extracts for all sub-classes, including sphingomyelins, ceramides, dihydroceramides, lactosylceramides and hexosylceramides. In the case of glycerolipids (MAGs, DAGs, and TAGs) the highest signals were obtained following single-step extraction with BUME and IPA, although the relative signal was not significantly different from the reference signal of the MTBE extract. Among glycerophospholipids, the significantly higher signal was obtained with IPA for PSs. For other subclasses the difference between three solvents was not significant. For lysophospholipids, the relative signal abundance was in general significantly higher for IPA and BUME extracts when compared to MTBE phase (Figure 2 and Figure S2, Tables S7 and S8).

To confirm these observations, six internal standards (IS), analogues for TAGs, PCs, LPCs, PEs, LPEs, and SMs, were spiked to the samples with the addition of organic solvent (see Figure S3). Based on relative signal abundance these internal standards demonstrated the similar tendency as the endogenous metabolites, depending on the lipid class. Globally, a higher signal was obtained for all different IS following IPA and BUME extraction compared to the organic MTBE phase from Matyash extraction, with the exception of TAG-d7, for which the signal was also the most variable (Figure S3).

In addition, in order to evaluate the extent of interference between lipids and polar metabolites when using one-step extraction coupled to HILIC-MS/MS analysis, the most abundant polar metabolites in human plasma (including different amino acids, organic acids, and acylcarnitines) were added to the MRM method for complex lipid analysis. We could appreciate that the selected polar metabolites, in the applied HILIC conditions, did not co-elute with complex lipids, therefore minimizing the potential effect of ion suppression on lipids (see Figures S4 and S5). Polar metabolites were effectively retained, but due to their high hydrophilicity, they eluted (with the exception of hydroquinone and hypoxanthine) after complex lipids in the chromatographic gradient.. Interestingly, similar results were found for the long chain acylcarnitines that did not co-elute with lipids, such as sphingomyelins when profiled in the positive ionization mode.

Finally, different organic solvents can lead to different contamination levels from plastic agents and, therefore, cause differences in matrix effects. To examine the potential influence of contaminants on lipid abundances, the background noise from blank extractions was examined using HRMS analysis (see Figure S6). Overall, no significant differences in the contaminant background were observed among the different solvent extracts, with the exception of MTBE extract profile in negative ionization mode, which showed several intense peaks eluting between 1 and 4 min. A few of these peaks likely represent fatty acids (stearic acid with *m*/*z* 283.26 and palmitic acid with *m*/*z* 255.23) that, in addition to being endogenous lipids, also represent the potential contaminants from plastics (confirmed with matching against MaConDa (https://www.maconda.bham.ac.uk/)).

#### 2.2.2. Polar Metabolite Profile

The same plasma extracts were analyzed for the detection and abundance of polar metabolites (as described in Materials and Methods). The HILIC-MS/MS method initially targeted 374 polar metabolites that were classified into 12 categories: proteinogenic amino acids, amino acid derivatives, nucleosides, nucleic acids, alkylamines, short and long chain acylcarnitines (SCACs and LCACs, respectively), carbohydrate conjugates, mono-, di-, and tri-carboxylic acids (Mono-COOHs, Di-COOHs, Tri-COOHs, respectively), and others (comprising glycocholate, hydroquinone, hydroxyphenyllactate, pyridoxine, salsolinol, trigonelline, and tryptamine, see Table S6 for more information). Similar to the complex lipid evaluation, we have evaluated the capacity of the BUME and IPA extractions methods to recover different polar metabolite classes with respect to signal abundance in the MeOH, as the reference method.

Significantly higher signals for the proteinogenic amino acids, carboxylic acids and carbohydrates were observed following the extraction with MeOH, while the nucleosides, alkylamines, short and long-chain acylcarnitines, and other polar metabolites showed significantly higher signals following the extraction with IPA, compared to BUME and MeOH (Figure 3 and Figure S7, Table S9). For amino acid derivatives and nucleic acids the signal intensity did not differ significantly between the MeOH, BUME, and IPA extracts.

Finally, the aqueous phase from biphasic Matyash extraction was also analyzed in order to evaluate the polar metabolite profile when compared to the profiles that were derived from other single-step methods (rich in lipids). For the majority of polar metabolites, with the exception of nucleic acids, amines, and alkylamines, the signal from the aqueous phase (following the Matyash protocol) was significantly lower when compared to single-step methods (Figure S8).

### 2.3. Reproducibility and Size of Measurable Lipidome and Metabolome

**Figure 4:**
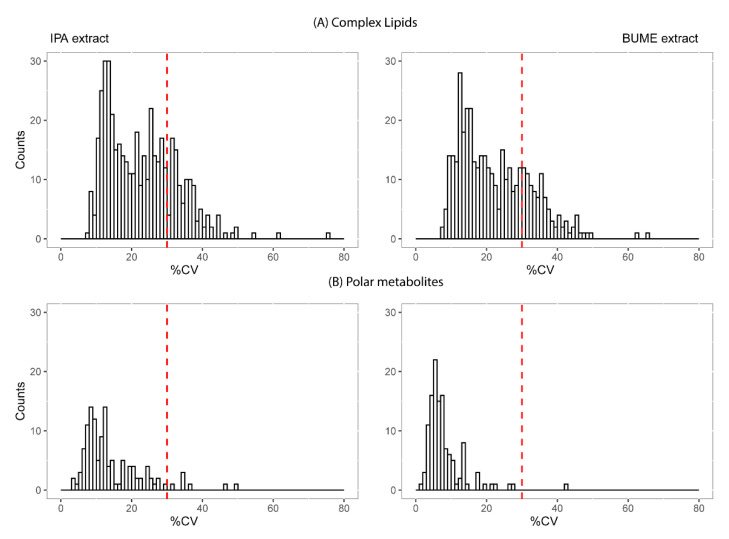
Signal reproducibility for complex lipids and polar metabolites. Coefficient of variation (CV) of all detected lipids (**A**) and polar metabolites (**B**) is presented in frequency histograms. The CV was evaluated across 20 independently prepared plasma samples (5 replicates × 4 different operators) using IPA and BUME. Red line indicates the threshold CV = 30% (data provided in Tables S10–S12).

The analytical variability of the selected single-step extraction methods using IPA and BUME was evaluated through independent preparation of pooled plasma samples by four different operators (*n* = 5 samples per operator and per solvent). The intra-batch coefficient of variation (CV) across 20 replicates, analyzed in a randomized fashion, was determined for both complex lipids and polar metabolites. Figure 4 represents the lipid and polar metabolite count, depending on the coefficient of variation across all replicates and operators (Tables S10–S12).

Out of 584 lipid species that were detected in pooled human plasma samples, 345 from the IPA extract had CV < 30% vs. 294 lipid species from the BUME extract (Figure 4A). The lipids with CV > 30% mainly included TAGs (169), ceramides (13, comprising DCER LCER, HCER), diacylglycerols (8), phosphatidylethanolamines (8), lysophosphatidylinositols (6), and cholesterol esters (6).

For polar metabolites, out of the 116 metabolites detected in pooled plasma samples, 109 from the IPA extract had a CV < 30% vs. 115 metabolites from the BUME extract (Figure 4B).

**Figure 5:**
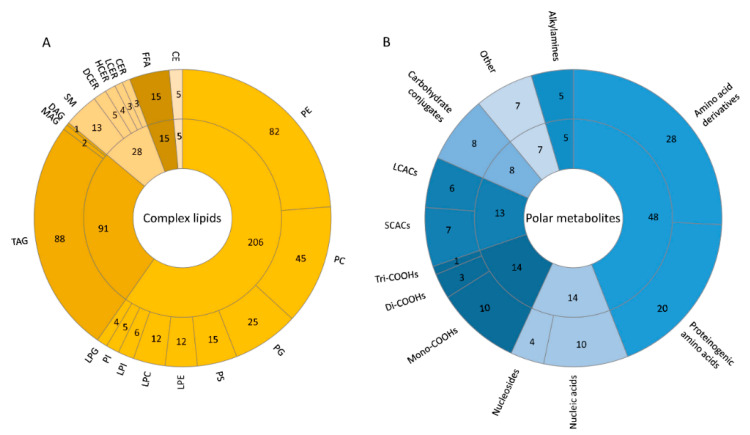
Diversity and size of measurable lipidome and polar metabolome. Complex lipids (**A**) and polar metabolites (**B**) that were reproducibly measured from IPA extract (CV < 30%, see Figure 4) are presented in the pie charts to show the broadest and most reproducible coverage. Lipid classification abbreviations: SM—sphingomyelin, CER—ceramides, DCER—dihydroceramides, LCER—lactosylceramiedes, HCER—hexosylceramides, MAG—monoacylglycerol, DAG—diacylglycerol, TAG—triacylglycerol, CE—cholesterol esters, FFA—free fatty acids, PI—phosphatidylinositol, PC—phosphatidylcholine, PG—phosphatidylglycerol, PE—phosphatidylethanolamine, PS—phosphatidylserine, LPC—lysophosphatidylcholine, LPG—lysophosphatidylglycerol, LPE—lysophosphatidylethanolamine, LPI—lysophosphatidylinositol, LPS—lysophosphatidylserine. Class abbreviations for polar metabolites: short (SCACs) and long chain (LCACs) acylcarnitines, mono- (Mono-COOHs)-, di- (Di-COOHs)-, and tri- (Tri-COOHs) carboxylic acids and others (comprising glycocholate, hydroquinone, hydroxyphenyllactate, pyridoxine, salsolinol, trigonelline, and tryptamine, see Table S6 for more information).

Following the filtering based on this analytical variability (CV < 30%), the size and diversity of the measurable lipidome and polar metabolome is shown in Figure 5 for the IPA extract, and in Figure S9 for the BUME extract.

## 3. Discussion

In this study, we have evaluated the performance of several single-step protocols for the simultaneous extraction of complex lipids and polar metabolites from the human plasma in a high-throughput manner. The extractions were performed with solvents that cover the following range of polarity indexes H_2_O > MeOH > EtOH > IPA > BuOH > MTBE, with the BUME mixture expected to be in the middle of this range [24,25]. Therefore, the extraction affinity of BUME is likely driven by the interaction of both butanol and methanol solvent, with the resulting polarity effect for the more hydrophobic (lipids) and hydrophilic (polar metabolites) compounds.

HILIC chromatography was chosen as the best compromise for performing complex lipid and polar metabolite analysis. It is important to note that different HILIC methods with different gradients and mobiles phases (see Materials and methods for details) were used for complex lipid and polar metabolite profiling, respectively. In addition to offering high chromatographic resolution for polar metabolite profiling, the HILIC approach is also advantageous for lipid analysis, since it allows for the separation of lipids classes based on their head groups. The separation by lipid class facilitates the development of quantitative methods, because each class can be covered by its corresponding internal standard. When compared to Reversed Phase (RPLC), the separation using HILIC also better corrects the matrix effects, since the endogenous lipids and their corresponding Internal Standards (IS) co-elute [26]. Importantly, HILIC separation also allows for the chromatography-assisted lipid quantification in large-scale population studies, due to the acceptable cost of a relatively low number of required internal standards.

The aim of our study was to evaluate whether the use of a single-step extraction generates the lipid and polar metabolite coverage equivalent to the commonly used biphasic protocols. To this end, we evaluated the abundance and repeatability of the MS signal of all detected endogenous lipids and polar metabolites in human plasma extracts instead of estimating the extraction efficiency of each solvent with the recovery assessment using pre- and post-extraction internal standard spike. Because complex lipids bind to protein carriers (i.e., lipoproteins), a property that cannot be reproduced by an internal standard spike, our strategy to evaluate the extraction method performance using the signal of extracted endogenous metabolites was considered as the best compromise. We argue that the best strategy to quantify the recovery of endogenous compounds is by using reference materials due to the complexity of biological matrices in general [27,28]. This approach is commonly applied for the validation of the measurement accuracy of analytical methods and was out of the scope of this study.

### 3.1. Complex Lipid Extraction

The extraction of lipids depends on their structure and substitute groups (i.e., headgroups that are representative of each lipid class), their polarity (determined by the head group and length of the alkyl chain) and their spatial configuration (e.g., degree of unsaturation). These structural characteristics play an important role in the interaction with the extraction solvent. As previously reported for BUME and IPA extraction, in general, more than 90% of the total lipid content of a plasma sample is extracted, and it is not deemed necessary to perform a re-extraction of the pellet [18,19].

Among different lipid classes, the relative signal abundance for sphingolipids was higher in IPA and BUME extracts when compared to MTBE. This can be explained by the polar character of the head group in the sphingolipid structure (Figure S10). The extraction of the sphingolipids with more polar character, such as lactosylceramides (LCer), was improved with more polar BUME mixture, owing to the lactosyl group. Conversely, in the case of hexosylceramides (HCER), significantly higher signal abundance was observed with IPA solvent as compared to MTBE and BUME (see Figure S11, showing the signal abundance of specific hexosylceramides and lactosylceramides with the same alkyl chain composition, across different solvents).

Glycerolipids and mono-, di-, and tri-alkyl substituted glycerols followed a similar trend of solvent affinity, MTBE < IPA < BUME, although this was observed without a statistically significant difference among the tested extraction methods. The arrangement of fatty acid chains located in any of three positions (sn-1/sn-2/sn-3) in the glycerol backbone may play an important role in the extraction affinity of this lipid class [29]. The highest affinity for BUME was the most pronounced for MAGs and DAGs having lower hydrophobicity, due to only one or two fatty acyl chains and the exposition of the hydroxyl group.

For cholesterol esters, which also have a highly lipophilic character similar to TAGs, no significant difference was observed between three extraction protocols, which could be due to the low intensity and high variability of signal due to their low electrospray ionization efficiency. The highest and the most reproducible response for these lipids was observed when using IPA as extraction solvent.

In the case of glycerophospholipids, the extraction appeared to be primarily driven by their hydrophobic character (due to their alkyl chains), since all subclasses were most efficiently recovered with the MTBE and IPA. These results, showing the strong affinity of PIs for the MTBE, and PGs, PCs and PEs for all three solvents, are in agreement with the previous reports of Lee et al. and Matyash et al. [17,30]. Importantly, the performance of the single-step extraction with IPA was at the same level as the reference method with MTBE for all glycerophospholipid classes. Even more, a significantly higher signal for PSs was obtained following the extraction with IPA (see Figure 2).

Finally, lysoglycerophospholipids, which are composed of a single fatty acyl chain, have lower hydrophobicity and, thus, their solubility mainly relies on the head group. Consequently, the extraction of lysophospholipids was more efficient with BUME and IPA. This effect was exacerbated for LPIs that were poorly recovered using the biphasic method with MTBE, thus limiting the LC-MS analysis of this class of lipids (i.e., signals close the level of detection).

### 3.2. Polar Metabolite Extraction

Large population studies require high-throughput methods, with minimal and highly reproducible sample preparation. Therefore, in this study, we investigated the viability of measuring polar metabolites from the same plasma extract that was obtained in single-step extraction used for the lipidome analysis. Polar metabolite analysis was performed using the optimized targeted HILIC-based methods (in acidic and basic conditions in positive and negative ionization mode, respectively) and methanol extraction was used as a reference for comparison [11,31].

Because BUME and IPA demonstrated the best performance for lipid extraction in a single step, we investigated their capacity to simultaneously extract polar metabolites implicated in central carbon metabolism (Table S9). Similar to lipid analysis, all of the polar metabolite classes were efficiently extracted by both methods. However, for the most polar classes, i.e., proteinogenic amino acids, carboxylic acids (mono-, di-, and tri-), and carbohydrate conjugates, the recovered signal was significantly lower while using IPA and BUME, as compared to methanol. Besides the hydrophilicity of these metabolites, the signal decrease following BUME and IPA extraction is also likely to be a consequence of ion suppression that is caused by co-eluting complex lipids (extracted with IPA and BUME) particularly in ESI positive mode, in the chromatographic conditions for polar metabolite analysis. Despite its decrease, the signal remained well defined and reproducible—a prerequisite for quantitative measurement.

For the majority of other polar metabolites and specifically nucleosides and alkylamines a significantly higher signal was observed with IPA when compared to BUME and MEOH. We hypothesize that this may be due to the representative heterocyclic compounds.

For acylcarnitines, the signal abundance varied, depending on the length of the acyl chain. Acylcarnitines are zwitterion molecules that are composed of a quaternary ammonium linked to a fatty acyl chain and their polarity varies, depending on the length of the acyl chain. The hydrophobic character of long-chain carnitines is evident, since the signal intensity was significantly higher in the IPA extract. For more polar short-chain acylcarnitines, the intensity of the recovered signal was also significantly higher in IPA extract but to a lesser extent. For both of these classes, methanol performance was significantly lower when compared to IPA.

It is important to note that when applying biphasic methods for lipid extraction, polar metabolites were previously reported to be successfully measured from the aqueous layer [32]. Interestingly, our results clearly showed the significantly lower signal abundance for the majority of polar metabolites following the biphasic Matyash protocol—as a result of the analysis of aqueous phase (when compared to BUME, IPA, and MEOH extracts, Figure S8). This observation is likely due to the partition of polar metabolites between the aqueous and organic phase in the Matyash extraction.

### 3.3. Extraction Method and Measurement Reproducibility

In terms of lipidome coverage, the single-step lipid extraction using IPA or BUME efficiently recovered all lipid sub-classes, which was already reported using untargeted approaches for the comparison of Matyash, Folch, Alshery (1:1, BUME), and IPA protocols [19,33,34,35]. Importantly, one-phase extraction using MMC solvent mixture (MeOH/MTBE/CHCl_3_) showed significantly better extraction efficiencies for moderate and highly non-polar lipid species in comparison with biphasic Folch, Bligh and Dyer, and MTBE extraction systems [32]. While the size of detectable lipidome was extensively explored in several previous studies, the relative abundance and repeatability of lipid signal was rarely evaluated. When evaluating the size of measurable lipidome and polar metabolome we observed a difference in lipid signal variability between IPA and BUME extracts following the reproducibility test (CV < 30% across independently extracted replicates). This difference was mainly due to the higher variability of specific lipid signals recovered from BUME extracts, comprising TAGs (20), some phospholipids, free fatty acids, and cholesterol esters. We hypothesize that the presence of MeOH and thus the mixture of two solvents, methanol, and butanol (*vs.* pure IPA) could contribute to this difference in CVs [36]. In general, high CVs that were observed for TAGs were likely caused by ion suppression as a consequence of high degree of co-elution in the void volume (using HILIC chromatography). The difference in terms of signal reproducibility was not observed for polar metabolites, likely thanks to a significantly higher degree of separation of hydrophilic metabolites and, thereby, the lower degree of co-elution throughout the chromatographic gradient.

The robustness of IPA and BUME extractions for lipidome analysis was previously assessed by independent studies using untargeted lipidomics approaches. For example, Calderón et al. compared the extraction methods while using CHCl_3_, MTBE, and IPA, after which IPA was reported as the most robust [37]. In another study, extraction protocols using MeOH, acetonitrile (ACN), IPA, IPA-ACN, CH_2_Cl_2_, CHCl_3_, MTBE, and Hexane were compared, and IPA was revealed as the best compromise to extract lipids [19]. In an additional study, where Folch, Matyash, and Alshery methods were tested, and the Alshery method was reported with high recoveries and lowest CV values for lipids [33]. All of these previous results highlight the differences in terms of signal reproducibility, depending on the lipid class and show that the most robust methods are the least biased single-step methods.

Beside robustness, the application of single-step plasma extraction in human population studies [38] is also advantageous in terms of high throughput. For instance, BUME extraction was applied to the study of differences in lipidome composition in the Singaporean population where three main communities, Chinese, Indian, and Malays were characterized [39]. It was also recently used in large-scale plasma lipidomic profiling to reveal the associations between lipid levels and cardiometabolic risk factors [40]. When compared to BUME that has been used in lipidomics community for more than 12 years now, the potential of IPA extraction for lipidome analysis was only recently revealed and it was therefore less commonly applied in lipidomics studies. However, several recent human population phenotyping studies warrant its application [19,41,42].

## 4. Materials and Methods

### 4.1. Chemicals and Reagents

Human pooled plasma samples were purchased from Sera Laboratories International Ltd. Trading as BioIVT (West Sussex, UK). A mixture of pooled male and female (40–60 years old) plasma was prepared and aliquoted for the extraction experiments.

Internal Standards, including sphingomyelin SM-18:1(d9) (1.96 µg/mL), triacylglycerol TAG-15:0/18:1/15:0(d7) (3.5 µg/mL), phosphatidylcholine PC-15:0/18:1(d7) (9.99 µg/mL), phosphatidylethanolamine PE-15:0/18:1(d7) (0.35 µg/mL), lysophosphatidylcholine LPC-18:1(d7) (1.58 µg/mL), and lysophosphatidylethanolamine LPE-18:1(d7) (0.32 µg/mL), were purchased from Avanti (Alabaster, AL, USA).

### 4.2. Metabolite Extraction Protocols

Human blood plasma (25 µL) was extracted with MeOH (125 µL), EtOH (125 µL), BUtanol:MEthanol (BUME, 125 µL), or isopropanol (IPA, 125 µL) that were pre-spiked with the above indicated mixture of internal standards in order to evaluate the performance of different extraction solvents. For the biphasic MTBE extraction 10 µL plasma was extracted with MTBE:MeOH:H_2_O (750/225/188 µL) [43]. Because plasma is homeostatically regulated no normalization to protein amount is required [44]. All of the samples were vortexed and kept at −20 °C for one hour to facilitate protein precipitation. These extracts were then centrifuged for 15 min at 20,000× *g* at 4 °C and the resulting supernatants, from MeOH, EtOH, BUME, and IPA, were collected and transferred to LC-MS vials for injection. The upper phase resulting from biphasic MTBE:MeOH:H_2_O extraction was evaporated to dryness (in a vacuum concentrator LabConco) and re-suspended in 50 µL of IPA for lipid extraction evaluation in order to maintain the same sample to solvent ratio (1/5), as in the single step extraction. Prior to LC-MS analysis, the extracted pooled plasma samples were randomized per operator and extraction solvent.

### 4.3. Protein Quantification

BCA Protein Assay Kit (Thermo Scientific, Waltham, MA, USA) was used in order to measure (A562nm) total protein concentration (Hidex, Turku, Finland). For this, the protein pellets were evaporated and reconstituted in the lysis buffer (20 mM Tris-HCl (pH 7.5), 4M guanidine hydrochloride, 150 mM NaCl, 1 mM Na_2_EDTA, 1 mM EGTA, 1% Triton, 2.5 mM sodium pyrophosphate, 1 mM beta-glycerophosphate, and 1 mM Na_3_VO_4_, 1 µg/mL leupeptin) using the Cryolys Precellys 24 sample Homogenizer (2 × 20 s at 10,000 rpm, Bertin Technologies, Rockville, MD, USA) with ceramic beads.

Protein quantification was performed on supernatant and plasma pellets in order to evaluate the efficiency of each solvent to precipitate proteins. The precipitation efficiency (Table S5) was calculated, as follows:

$$\mathrm{Protein}\mathrm{precipitation}\mathrm{efficiency}=\frac{protein\;content\;in\;plasma\;pellet}{protein\;content\;in\;plasma\;pellet+supernatant\;}\times \;100$$

### 4.4. LC-MS/MS Analysis

#### 4.4.1. High-Coverage Targeted Lipidome Analysis

For the lipidome analysis, the extracted plasma samples were analyzed by Hydrophilic Interaction Liquid Chromatography coupled to tandem mass spectrometry (HILIC-MS/MS) in positive and negative ionization modes using a Q-TRAP 6500 plus LC-MS/MS system (Sciex, Darmstadt, Germany). In both positive and negative ionization mode, the chromatographic separation was carried out on an Acquity BEH Amide, 1.7 µm, 100 mm × 2.1 mm I.D. column (Waters, Milford, MA, USA). Mobile phase was composed of A = 10 mM ammonium acetate in Acetonitrile:H_2_O (95:5) (pH = 8.2) and B = 10 mM ammonium acetate in Acetonitrile:H_2_O (50:50) (pH = 7.4). The linear gradient elution from 0.1% to 20% B was applied for 2 min, from 20% to 80% B for 3 min, followed by 3 min of re-equilibration to the initial chromatographic conditions. The flow rate was 600 µL/min, column temperature 45 °C, and sample injection volume 3 µL. Optimized ESI Ion Drive Turbo V source parameters were set, as follows: Ion Spray (IS) voltage 5500 V in positive mode and −4500 V in negative mode, curtain gas 35 psi, nebulizer gas (GS1) 50 psi, auxiliary gas (GS2) 60 psi, and source temperature 550 °C. Nitrogen was used as the nebulizer and collision gas. Optimized compound-dependent parameters were used for data acquisition in scheduled multiple reaction monitoring (sMRM) mode. Transitions for the entire panel of targeted lipids were optimized by SCIEX while using the Lipidyzer™ Platform [45]. The pooled plasma sample was first analyzed by MRM (non-scheduled) in order to obtain the retention time of each lipid class in the applied chromatographic system and added later to the scheduled MRM method (Tables S1 and S2).

#### 4.4.2. High-Coverage Targeted Metabolome Analysis

For the polar metabolome analysis, the extracted plasma samples were analyzed by HILIC-MS/MS in both positive and negative ionization modes while using a 6495 triple quadrupole system (QqQ) interfaced with 1290 UHPLC system (Agilent Technologies, Santa Clara, CA, USA). In the positive mode, the chromatographic separation was carried out in an Acquity BEH Amide, 1.7 µm, 100 mm × 2.1 mm I.D. column (Waters, MA, USA). Mobile phase was composed of A = 20 mM ammonium formate and 0.1% FA in water and B = 0.1% formic acid in ACN. The linear gradient elution from 95% B (0–1.5 min) down to 45% B was applied (from 1.5 to 17 min) and these conditions were held for 2 min. Subsequently, initial chromatographic condition were maintained as a post-run during 5 min for column re-equilibration. The flow rate was 400 µL/min, column temperature 25 °C, and sample injection volume 2 µL. In negative mode, a SeQuant ZIC-pHILIC (100 mm, 2.1 mm I.D., and 5 µm particle size (Merck, Darmstadt, Germany) column was used. The mobile phase was composed of A = 20 mM ammonium Acetate and 20 mM NH_4_OH in water at pH 9.7 and B = 100% ACN. The linear gradient elution from 90% (0–1.5 min) to 50% B (8–11 min) down to 45% B (12–15 min). Finally, the initial chromatographic conditions were established as a post-run during 9 min for column re-equilibration. The flow rate was 300 µL/min, column temperature 30 °C, and sample injection volume 2 µL.

ESI source conditions were set, as follows: dry gas temperature 290 °C, nebulizer 35 psi and flow 14 L/min, sheath gas temperature 350 °C and flow 12 L/min, nozzle voltage 0 V, and capillary voltage +2000 V and −2000 V in positive and negative mode, respectively. Dynamic Multiple Reaction Monitoring (DMRM) was used as an acquisition mode with a total cycle time of 600 ms. The MRM transitions were optimized from the direct analysis of pure chemical standards that were obtained from Sigma–Aldrich (The Mass Spectrometry Metabolite Library of Standards—MSMLS) (Tables S3 and S4). These transitions are publicly available in the Metlin-MRM spectral library [46].

### 4.5. Data (Pre)Processing

Raw LC-MS/MS lipidome data were processed (i.e., peak extraction and alignment) while using the Multi Quant Software (version 3.0.3, Sciex, Framingham, MA, USA) and raw metabolome data was processed using the Agilent Quantitative analysis software (version B.07.00, MassHunter, Agilent technologies, Santa Clara, CA, USA). The data on peak height and peak area were extracted for each lipid and polar metabolite based on its extracted ion chromatograms (EICs) for the monitored MRM transitions. The peaks were filtered based on their presence (in 100% of replicates across all operators) and the intensity threshold (of 5 × 10^3^ ion counts), and the obtained tables (containing peak areas of detected metabolites across all replicates) were exported to R version 3.5.1 software http://cran.r-project.org/ and RStudio version 1.1.463. For a quality control, the peaks were filtered based on their coefficient of variation calculated per solvent, while using the independent replicates analyzed across the entire run, when considering all four operators. Peaks with CV > 30% were removed from further analysis with the aim to determine the size of measurable and not only detectable lipidome and metabolome.

### 4.6. Statistical Data Analysis

R packages “tidyverse” and “ggplot2” were used to format the data and plot the figures, respectively. Fold changes were calculated while using the peak area values (no transformation and/or scaling was applied). GraphPad Prism 6 (GraphPad Software Inc., La Jolla, CA, USA) was used for statistical data analysis. One-way ANOVA was used in order to test the significance of signal abundance between replicates that were extracted with different solvents with an arbitrary level of significance *p*-value = 0.05.

## 5. Conclusions

Two single-step sample preparation methods, using IPA and BUME, were evaluated as the best compromise for the simultaneous and reproducible extraction of complex lipids and polar metabolites from human plasma. The relative signal abundance of complex lipids in IPA and BUME extracts was greater or equivalent to the signal that was recovered with the Matyash method using MTBE (commonly applied in lipidomics). Importantly, the MTBE showed limited performance for the extraction of lysophospholipids, and particularly lysophosphatidylinositols (LPIs). Although the breadth of coverage was the same for both single-step methods, the most robust lipid profiling was achieved following IPA extraction with the greatest number of profiled lipids with CV < 30%, and specifically TAGs. In addition to complex lipids, both IPA and BUME method extracted polar metabolites successfully, but less efficiently than methanol. These polar metabolites included proteinogenic amino acids and acylcarnitines, di- and tri-carboxylic acids, carbohydrates, and nucleosides. Based on the examined lipidome and polar metabolome coverage, extraction efficiencies, effectiveness of protein precipitation, and reproducibility, we conclude that both methods, IPA and BUME, are suitable for merged lipid and polar metabolite analysis in large-scale human population studies.

## Supplementary Materials

The following supplementary figures and tables are available online at https://www.mdpi.com/2218-1989/10/12/495/s1: Figure S1. Relative signal abundance of different lipid classes in MeOH, EtOH, IPA and BUME extracts of human plasma, Figure S2. Relative signal abundance (per lipid class) to signal recovered with biphasic extraction using MTBE, Figure S3. Signal abundance of internal standards (representing six lipid classes) spiked into plasma samples during single step IPA, BUME and biphasic Matyash extraction (organic MTBE phase), Figure S4. Retention of polar metabolites and complex lipids throughout the chromatographic gradient applied for complex lipid analysis using HILIC-MS/MS in positive ionization mode, Figure S5. Retention of polar metabolites and complex lipids throughout the chromatographic gradient applied for complex lipid analysis using HILIC-MS/MS in negative ionization mode, Figure S6. Background noise from blank extractions performed with (A) methanol, (B) Matyash extraction (organic MTBE phase), (C) IPA and (D) BUME, Figure S7. Relative signal abundance (per polar metabolite class) to signal recovered with MeOH extract, Figure S8. Relative signal abundance of polar metabolites detected in the aqueous phase of Matyash extraction compared to other extraction protocols (including methanol extract as a reference), Figure S9. Diversity and size of measurable lipidome and polar metabolome from BUME extract, Figure S10. Signal intensity of the sphingomyelins detected in the method, Figure S11. Signal intensity of specific hexosylceramides and lactosylceramides with the same alkyl chain composition in IPA, BUME and MTBE extracts, Table S1. MRM transitions of lipids in positive mode, Table S2. MRM transitions of lipids in negative mode, Table S3. MRM transitions of polar metabolites in positive mode, Table S4. MRM transitions of polar metabolites in negative mode, Table S5. Protein content per each solvent or solvent mixture, Table S6. Classification of polar metabolites, Table S7. Abundances (peak areas) of lipid species detected by the HILIC-MS/MS analysis of each solvent extract (organic MTBE phase from Matyash protocol, IPA and BUME) in positive ESI mode, Table S8. Abundances of lipid species detected by the HILIC-MS/MS analysis of each solvent extract (organic MTBE phase from Matyash protocol, IPA and BUME) in negative ESI mode, Table S9. Abundances (peak areas) of polar metabolites detected by the HILIC-MS/MS analysis of each solvent extract (methanol, IPA and BUME) in positive and negative ESI modes, Table S10. Abundances and CVs of lipid species detected by the HILIC-MS/MS analysis of selected single-step extractions (IPA and BUME) in positive ESI mode, Table S11. Abundances and CVs of lipid species detected by the HILIC-MS/MS analysis of selected single-step extractions (IPA and BUME) in negative ESI mode, Table S12. Abundances and CVs of polar metabolites detected by the HILIC-MS/MS analysis of selected single-step extractions (IPA and BUME) in positive and negative ESI mode.
